# The Effect of Evaluating Perfusion with Infrared Fluorescent Angiography on Flap Survival in Head and Neck Free Flap Reconstruction

**DOI:** 10.3390/diseases12100255

**Published:** 2024-10-16

**Authors:** Ayten Saracoglu, Gamze Tanirgan Cabakli, Kemal Tolga Saracoglu, Gul Cakmak, Ilhan Erdem, Tumay Umuroglu, Bulent Sacak, Pawel Ratajczyk

**Affiliations:** 1 Department of Anaesthesiology, ICU, and Perioperative Medicine, Hamad Medical Corporation, Doha P.O. Box 3050, Qatar; asaracoglu@hamad.qa (A.S.); ksaracoglu@hamad.qa (K.T.S.); 2 College of Medicine, QU Health, Qatar University, Doha P.O. Box 2713, Qatar; 3 Department of Anesthesiology and Reanimation, Marmara University School of Medicine, Maltepe 34854, Türkiye; drgulcakmak@gmail.com (G.C.); tumay.oncel@marmara.edu.tr (T.U.); 4 Department of Plastic and Reconstructive Surgery, Marmara University School of Medicine, Maltepe 34854, Türkiye; ilhanerdemmd@gmail.com (I.E.); bulent.sacak@acibadem.com (B.S.); 5 Department of Anesthesiology and Intensive Therapy, Medical University of Lodz, 90-151 Lodz, Poland

**Keywords:** fluid management, free flap, spy, microcirculation

## Abstract

Introduction: Intraoperative fluid management is one of the most important factors affecting optimal perfusion in the microcirculatory area in patients that undergo flap surgery. While insufficient fluid administration in the intraoperative period leads to flap complications and organ dysfunction, volume load can cause complications such as edema in the denervated flap tissue, the opening of the sutures, or fat necrosis. The Infrared Fluorescent Angiography Perfusion Evaluation Device (SPY) is one of the many noninvasive techniques that evaluate the well-being of microcirculation at the tissue level. This device monitors and scores the perfusion distribution in the flap area. This retrospective study aimed to investigate the effect of fluid resuscitation in head and neck free flap transfer surgery on flap quality and patient outcomes according to the change in SPY scores. Material and Method: This study included 39 ASA I–II patients who were aged 18–60 years and underwent simultaneous free flap reconstruction of the head and neck between 2015 and 2021. Patients’ blood pressure, body temperature, hemoglobin, pH, and lactate values were recorded at both baseline and end of the operation. Also, the SPY “Infrared Fluorescent Angiography Perfusion Evaluation Device” scores, the amount of intraoperative fluid and transfusion, bleeding and urine output, and the duration of mechanical ventilation, anesthesia and surgery, and the duration and amount of drainage, the length of stay in hospital and intensive care unit, and the presence of flap infection, detachment, necrosis and loss, and re-exploration rate were recorded for the patients. Results: The difference between the first and last measured SPY values was observed to be positively correlated with the length of stay in the hospital and intensive care unit and the duration of drainage. There was a positive correlation between the length of stay in the hospital and intensive care unit and the duration of drainage, the amount of drainage, as well as the duration of anesthesia and the duration of surgery (*p* < 0.001). A positive correlation was found between the amount of drainage and the amount of crystalloid solution administered (r = 0.36, *p* < 0.05). In patients with flap infection, the difference between SPYfirst and SPYlast, the duration of anesthesia, and the duration of surgery were significantly higher. The amount of crystalloid solution given and bleeding and the duration of anesthesia and surgery were found to be significantly higher in mechanically ventilated patients (*p* < 0.05). Conclusions: It has been concluded that SPY-guided fluid management can be beneficial in preventing morbidities, such as extended hospital and intensive care stay, by reducing flap infection, mechanical ventilation duration, and drainage, with early diagnosis of insufficient perfusion.

## 1. Introduction

Optimization of macrocirculation does not always provide accurate information about the quality of true tissue perfusion and oxygenation. A previous study demonstrated that although the macrocirculatory results of the patient improved after traumatic hemorrhagic shock, the impairment of the microcirculation level continued for 72 h [1]. The use of microcirculatory perfusion parameters can improve free flap results. For this purpose, many methods are used that allow the monitoring of microcirculation in the tissue, and SPY is one of the devices used to monitor the perfusion of the relevant tissue with near-infrared fluorescence imaging [2]. Recently, the use of fluorescence imaging was found to be safe for rapid screening of infections resistant to lactam antibiotics [3]. Zhang et al. [4] tried the fluorescent probe successfully to detect nitric oxide in living cells of an inflammatory model.

However, in clinical practice today, conventional liberal fluid therapy is still preferred in patients to reduce and manage surgical losses during free flap surgery [5]. Blind and estimated fluid calculations are used, taking into account the width of the surgical incision area. Very few centers started to use intraoperative algorithms based on fluid responsiveness and cardiac output measurements on patients who are to undergo major surgery. However, even all these parameters can enable patients to undergo an operation with the guidance of macrocirculatory parameters with targeted fluid therapy [6]. Microcirculatory-guided fluid therapy, in which algorithms based on microcirculatory data are used, is performed on a very limited scale, although it is recommended in published guidelines [7,8].

The primary purpose of this retrospective study is to examine the effects of changes in SPY scores on flap quality and patient outcomes in head and neck free flap transfer surgeries. The secondary purpose is to detect surgical and medical complications.

## 2. Material and Method

In this retrospective cross-sectional study, obtained from the hospital database, the electronic data of 39 patients with ASA I–II who were aged between 18 and 60 years and underwent breast free flap reconstruction between January and December 2022 were included in the study following the approval of the local ethics committee. The patients with ASA III–IV, those with known neurological or psychiatric disorders, those with a clinically significant cardiovascular, respiratory, hepatic, renal, or metabolic disease, those with long-term drug or alcohol dependence and diabetes mellitus, those whose BMI > 30 and age > 60, mentally challenged patients, and those with a history of massive bleeding, coagulopathy, hypothermia, and unexpected intraoperative surgical complications were excluded from the study.

### 2.1. SPY Measurements

The near-infrared (NIR) light coming from the imaging module is projected on the patient through a fiberoptic cable so that the NIR excitation light in the imaging headpiece comes over and illuminates the relevant area. The patient is given “indocyanine green” (Indocyanine Green-ICG), the ICG is bound to the plasma proteins in the blood and sent to the relevant area of the blood circulation, and the ICG is made to emit fluorescent light with the NIR excitation light emitted by the imaging headpiece of the device. This is detected by an NIR camera, and the resulting image signal is processed and recorded on a computer. It is also displayed in real-time on video monitors [1]. The data obtained from the SPY image recordings were recorded.

### 2.2. Fluid Therapy

The fluid management of the patients was performed at 6–8 mL/kg/h with crystalloid fluids in line with the fluid resuscitation regimen, which is designed by the relevant institution and applied by an authorized anesthesiologist in such surgeries. Hydroxyethyl Starch (HES), a colloid liquid, was administered if signs of hypovolemia developed. A decrease of more than 20% in the mean arterial pressure relative to the baseline value is considered as hypotension in our clinic. In flap surgeries, the target hemoglobin value is 10 g/dL and blood transfusion is performed when necessary.

### 2.3. Monitoring

A standard monitoring method including electrocardiogram, peripheral oxygen saturation, heart rate, respiratory rate, temperature, and end-tidal CO_2_ pressure measurement is applied to the patients. Expiratory CO_2_ concentrations are maintained between 30 and 35 mmHg. Baseline Bispectral Index (BIS) values in the awake state are recorded with the eyes closed 1–2 min before the induction of anesthesia. Then, anesthesia is induced by administering propofol 1–2 mg/kg, rocuronium 0.6 mg/kg, and fentanyl 2–3 mcg/kg. For the maintenance anesthesia of the patient, an inhalation anesthetic agent is provided with 3 lt/min flow and remifentanil 0.2 mcg/kg/min infusion in 80% O_2_ and 20% air. A Primus (Draeger, Lubeck, Germany) anesthesia device is used during the maintenance of anesthesia. If necessary, the maintenance of muscle relaxation is ensured with additional rocuronium. Following the operation, muscle relaxation is restored with neostigmine 0.05 mg/kg iv and atropine 0.015 mg/kg iv. Extubation is performed when patients’ TOF is >90%. The time elapses until the modified Aldrete score is 9–10 and the total additional dose of remifentanil is recorded.

Obtained from patient records, hemodynamic variables such as systolic blood pressure (SBP), diastolic blood pressure (DBP), mean arterial pressure (MAP), heart rate (HR), and oxygen saturation (SaO_2_) were recorded. Operation and anesthesia duration, gender, age, BMI (kg/m^2^), presence of DM/HT, ASA score, amount of intraoperative fluid and blood transfusion, ischemia duration, blood loss, urine output, vasopressor use, SPY scores, lactate in arterial blood gas, HCO_3_, and pH values were recorded.

In the postoperative period, the patients’ urine output, acute renal failure, myocardial infarction (MI), deep vein thrombosis, cerebrovascular accident, sepsis, presence of pneumonia, complete or partial flap loss on day 30, wound dehiscence, fat necrosis, infection of the flap and/or donor site, re-exploration, length of stay in the intensive care unit, length of hospital stay, duration of mechanical ventilation, and need for dialysis were recorded.

### 2.4. Statistical Analysis

SPSS version 23.0 (IBM Corp., Armonk, NY, USA) was used in all statistical analyses. The data were presented as mean ± SD for normally distributed continuous variables, and as median (25–75%) for non-normally distributed continuous variables. An ANOVA test was used in the analysis of normally distributed data, and Repeated Measure ANOVA analysis was used in that of repetitive data. Student’s *t* test was used in the analysis of normally distributed data, and Repeated Measure ANOVA analysis was used in that of repetitive data. Correlation analyses were conducted with Pearson and Spearman correlation methods.

Assuming that the difference in SPY values between the groups would be 25% and the standard effect size would be 1, it was found sufficient to include 20 cases in each group. *p*-value < 0.05 was considered statistically significant.

## 3. Results

**Figure 1:**
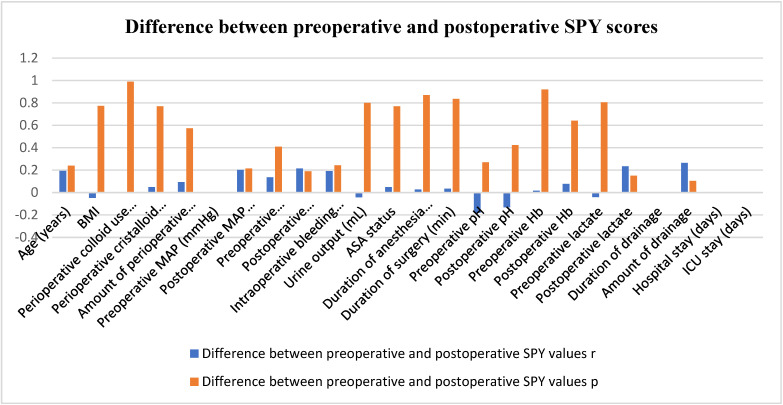
The relationship between the duration and the amount of drainage, the length of hospital and the intensive care unit stay, and the blood gas parameters.

The mean age of the patients was 55.13 ± 20.066 years (Table 1). The preoperative SPY value was 66.687 ± 24.5406, and the postoperative SPY value was 68.450 ± 28.0438. The difference between preoperative and postoperative SPY values was −1.7628 ± 17.2796, and a significantly positive correlation was found between preoperative MAP values (r = 0.32, *p* < 0.05), drain duration (r = 0.45, *p* < 0.01), length of hospital stay (r = 0.41, *p* < 0.01), and length of stay in the intensive care unit (r = 0.32, *p* < 0.05) (Figure 1).

There was a positive correlation between the amount of drainage and the length of hospital stay (r = 0.56, *p* < 0.001), the length of stay in the intensive care unit (r = 0.44, *p* < 0.01), and the amount of intraoperative crystalloid (r = 0.36, *p* < 0.05) (Table 2).

There was also a positive correlation between the length of hospital stay and the postoperative pH values (r = 0.36, *p* < 0.05), the anesthesia duration (r = 0.43, *p* < 0.01), and the operation duration (r = 0.45, *p* < 0.01) (Table 2). Another positive correlation was found between the length of stay in the intensive care unit, the anesthesia duration (r = 0.33, *p* < 0.05), and the operation duration (r = 0.33, *p* < 0.05) (Table 2).

The difference between preoperative and postoperative SPY values in the patients with a flap or donor infection was significantly higher than those without infection (*p* = 0.008, Table 3). The anesthesia and operation duration were significantly longer in the patients with a flap or donor infection than those without infection (*p* = 0.012, *p* = 0.017, respectively).

The difference between preoperative and postoperative SPY values was found to be significantly higher in the patients with wound dehiscence compared to those without (*p* < 0.001, Table 4). The amount of perioperative crystalloid infusion, the anesthesia duration, the operation duration, and the amount of bleeding was found to be significantly higher in the patients who received postoperative mechanical ventilation support (*p* = 0.034, *p* = 0.034, *p* = 0.032, *p* = 0.006, respectively, Table 5).

## 4. Discussion

Our study aimed to investigate the effects of preoperative and postoperative SPY values in patients having undergone free flap reconstruction surgery on patient and graft survival. It was revealed that a change in SPY values is positively correlated with drain duration and length of stay in hospital and intensive care units. In addition, the change in SPY values was found to be significantly higher in the patients with wound dehiscence, flap, or donor infection.

In our study, the difference between BMI, age, ASA score, lactate values, urine output, postoperative mean arterial pressure, baseline and postoperative temperature values, total intraoperative bleeding, the amounts of colloid fluid and blood transfusion, and SPY values did not indicate a significant correlation. This suggests that it might be related to the small sample size of our study.

Our results show that the increase in the measured baseline mean arterial pressure and the difference between the SPY values are correlated (r = 0.32, *p* < 0.05), which indicates that the blood pressure control in these patients in the preoperative period may have a significant effect on ensuring adequate tissue perfusion in the intraoperative period.

Total flap loss occurs at a rate of 4–10% in free flap surgery, and partial necrosis develops in many patients [9]. Even if the cause is a surgical procedure, a disorder in microcirculation, which is the final pathway of cellular oxygen and substrate distribution, may develop after tissue trauma, and this can both deteriorate flap health and lead to a process that can result in multi-organ failure in the postoperative period. Therefore, it is crucial to monitor tissue perfusion in reconstructive surgeries. In addition, positive fluid balance can severely damage flap tissue as a result of edema. It is known that flap tissue is at risk of developing edema due to denervation and lymphatic deficiency. The significant correlation found in our study between the amount of postoperative drainage and the amount of intraoperative crystalloid fluid administered (r = 0.36, *p* < 0.05) was interpreted as a result of maintaining the well-being in macrocirculatory parameters through blind methods and administering fluid to each patient in the same way. In addition, evidence-based use of vasoactive drugs used in the intraoperative period is recommended, since they may trigger a spasm in the anastomosis and lead to a decrease in graft perfusion and ultimately flap failure [10]. Kass et al. [11] examined 445 patients who underwent head and neck free flap reconstruction and concluded that intraoperative fluid resuscitation with a large volume of more than 5 L was associated with an increased incidence of free flap loss. Another study showed that postoperative complications such as wound dehiscence and flap failure increase with the perioperative administration of large amounts of intravenous fluid [12]. Evidence-based recommendations suggest that fluid management in the intraoperative period should not go beyond the range of 3.5–6 mL/kg/h [13]. In our study, different anesthesiologists adjusted fluid infusions according to their own clinical experience, and fluid was not administered in volumes higher than 8 mL/kg/h. According to our results, the higher amount of intraoperative crystalloid infusion in patients receiving postoperative mechanical ventilation support reveals the importance of indicators that can globally indicate microcirculatory tissue perfusion. It was also found in our study that the amount of bleeding in the patients who received postoperative mechanical ventilation support was significantly higher in the intraoperative period.

A crucial factor in any clinical study is the selection of patients, as this can introduce bias, which may significantly influence the results. Bias, particularly in patient selection, can distort the relationship between the variables being studied and the outcomes. For instance, if the selection process inadvertently favors patients with specific characteristics, the findings may not be representative of the broader population undergoing free flap reconstruction surgery. In our study, patient selection was based on retrospective data, which inherently carries the risk of selection bias. This could potentially affect the generalizability of our findings, particularly regarding the correlation between SPY scores and clinical outcomes such as drain duration, length of stay, and wound complications.

To mitigate the impact of bias on study results, we recommend considering advanced statistical techniques such as propensity score matching or other methods designed to adjust for potential confounders. These methods could help to account for differences in baseline characteristics like BMI, age, or ASA score, which, although not significantly correlated with SPY values in our study, might still influence the outcomes in larger or more diverse cohorts. In our study, we found that changes in SPY scores were positively correlated with the duration of postoperative drainage, length of hospital stay, and the development of complications such as wound dehiscence, flap infection, or donor site infection. These findings support the importance of close monitoring of tissue perfusion in patients undergoing free flap reconstruction. However, we did not find significant correlations between SPY values and variables such as BMI, age, ASA score, or other intraoperative parameters, which may be attributable to the relatively small sample size. Larger studies are required to verify these relationships and reduce the potential for type II errors.

In our study, the mean duration of surgery was found to be 466.67 ± 119.513 min. In a study conducted by The American College of Surgeons National Surgical Quality Improvement Program (ACS NSQIP) and involving 108.303 patients, it was revealed that flap-related complications and thus the risk of reoperation increase if the operation lasts longer than 10 h [14]. In a similar vein, it was observed in this study that the anesthesia and operation duration were longer in the patients with a flap or donor infection. The results also demonstrate that an increase in anesthesia and operation duration increases the length of stay in the intensive care unit and hospital. This emphasizes that the widespread use of monitoring methods during free tissue transfer can improve patient outcomes by shortening the duration of operation and therefore that of anesthesia.

Many monitoring methods have been developed that allow the examination of microcirculatory perfusion markers at the tissue level. The concept of assessing the success of resuscitation through assessing hemodynamic adaptation, on the other hand, refers to the compatibility of this condition with macrocirculatory perfusion. It is also disquieting that the improvement in the parameters that shed light on the macrocirculatory status of the patients does not always accompany the improvement in the perfusion in the microcirculatory area [15]. In our study, quantitative analysis of fluorescent imaging recorded with a SPY device, which is one of these methods, was performed. These measurements performed in the surgical field were recorded in the preoperative and postoperative periods. In recent years, this method has begun to be used more frequently during the intraoperative period in different types of surgery [16]. Considering that the earlier the existing ischemia is detected, the more possible it is to save the flap, the importance of this monitoring method becomes evident. Our results show that the higher SPY score in patients with a flap or donor infection and wound dehiscence also manifests the necessity of flap perfusion monitoring.

In the study that Dasta et al. [17] conducted, a daily average cost of USD 3436 is spent after the second day of stay in the intensive care unit. Therefore, the fact that the difference between the preoperative and postoperative SPY scores is related to the length of stay in the intensive care unit clearly shows that tissue perfusion monitoring will also contribute to reducing financial costs by enabling us to gain insights into the length of stay in both hospital and intensive care unit. In addition, it might provide a preliminary idea about wound site infection or separation. In light of these results, we believe that this type of monitoring will offer insights into the decisions taken regarding the postoperative follow-up of patients in whom there is a significant difference between the first and last measured SPY scores for surgery.

It is possible to evaluate micro anastomoses and tissue transplants in the intraoperative period thanks to SPY [18]. Massaro et al. [19] reported that a patient who underwent intraoperative perfusion measurement with SPY could be reoperated before leaving the operating room. There were not any patients for whom a decision for reoperation or revision was made in our study, which reveals that SPY follow-up will lead to significant gains in patient survival. We believe that this imaging technique will become the gold standard in determining regional tissue perfusion saturation as the number of studies in this field increases.

### Limitations

In recent years, the administration of intravenous fluids and vasopressors to optimize tissue perfusion, reduce complications, and improve patient outcomes has begun to be implemented under the guidance of goal-directed therapy (GDT) that is individualized with minimally invasive hemodynamic monitors [20]. For this purpose, various invasive and non-invasive cardiac output measurement devices are used. Its presence can provide additional information concerning fluid balance. In addition, the benefits of fluid resuscitation applications conducted based on tissue perfusion measurements with microcirculatory cameras are known in the follow-up of perfusion parameters. However, the results of these imaging methods could not be used in our study because they were not available in our clinic. A correlation was found between the difference in SPY scores and both the length of hospital stay and the duration of drainage. However, a long hospital stay does not usually mean a long drainage time in clinical practice. There are several affecting factors. Flap edema or infection are among the leading causes for a prolonged hospital stay. Another limitation is that the study was designed retrospectively. Prospective studies with increased sample sizes are needed.

## 5. Conclusions

We conclude that SPY-guided fluid management can be beneficial in preventing complications such as flap infection and wound dehiscence by diagnosing insufficient perfusion early, and in preventing morbidities such as increased hospital and intensive care unit stays by reducing mechanical ventilation duration and postoperative drainage.

## Figures and Tables

**Table 1:** Analysis of patients’ demographic data, spy values, and intraoperative variables.

	Mean	Std. Deviation
Age (years)	55.13	20.066
BMI	25.63	23.45
Preoperative Spy	66.687	24.5406
Postoperative spy	68.450	28.0438
Amount of perioperative transfusion (mL)	332.05	62.9064
Perioperative cristalloid use (mL)	4551.28	1907.861
Perioperative colloid use (mL)	733.33	409.964
Duration of anesthesia (min)	477.56	121.357
Duration of surgery (min)	466.67	119.513
Preoperative MAP (mmHg)	98.28	11.507
Postoperative MAP (mmHg)	84.56	10.845
Preoperative temperature (°C)	36.51	0.526
Postoperative temperature (°C)	36.59	0.604
Intraoperative bleeding (mL)	764.10	764.074
Urine output (mL)	1095.90	750.911
Preoperative pH	7.4497	0.03572
Postoperative pH	7.4374	0.04121
Preoperative lactate	1.3436	0.56279
Postoperative lactate	1.6692	0.90355
Difference between preoperative and postoperative SPY	−1.7628	17.27960

**Table 2:** The relationship between the duration and the amount of drainage, the length of hospital and the intensive care unit stay, and the blood gas parameters.

	Duration of Drainage		Amount of Drainage		Hospital Stay (Days)		ICU Stay (Days)	
Hospital stay (days)	0.694	0.000	0.564	0.000				
ICU stay (days)	0.571	0.000	0.443	0.005				
Amount of perioperative transfusion (mL)	0.200	0.223	0.242	0.138	0.165	0.315	0.235	0.151
Perioperative cristalloid use (mL)	0.300	0.063	0.365	0.022	0.190	0.247	0.213	0.192
Perioperative colloid use (mL)	0.120	0.465	0.156	0.344	0.025	0.881	0.096	0.560
Postoperative lactate	0.056	0.737	−0.095	0.565	−0.079	0.632	0.022	0.896
Postoperative Hb	−0.072	0.661	−0.092	0.577	−0.206	0.209	−0.121	0.464
Postoperative pH	0.350	0.029	0.293	0.070	0.362	0.023	0.238	0.144
Postoperative temperature (°C)	0.109	0.507	−0.023	0.889	−0.034	0.836	0.038	0.818
Postoperative MAP (mmHg)	−0.024	0.884	−0.011	0.947	−0.023	0.890	−0.094	0.570
Duration of anesthesia (min)	0.293	0.070	0.290	0.073	0.435	0.006	0.336	0.037
Duration of surgery (min)	0.278	0.086	0.282	0.082	0.453	0.004	0.335	0.037
ASA status	0.158	0.336	0.270	0.097	0.208	0.204	0.218	0.182
Age (years)	0.022	0.896	0.028	0.866	0.124	0.453	0.115	0.484
BMI	0.107	0.517	0.088	0.595	−0.041	0.803	0.212	0.195

**Table 3:** Analysis of the relationship between flap or donor infection and spy values, the amount of blood and fluid transfusion, the duration of surgery, and blood gas parameters.

Flap or Donor Infection					
	Mean	SD	Mean	SD	*p*
Preoperative SPY score	63.68	21.855	78.35	32.041	0.253
Postoperative SPY score	70.78	21.212	59.44	47.162	0.527
Difference between preoperative and postoperative SPY scores	−7.10	8.057	18.91	26.983	0.008
Fluid balance for 48 h	1397.10	520.578	1075.00	781.482	0.299
Amount of perioperative transfusion	345.16	688.641	281.25	334.811	0.712
Perioperative cristalloid use	4306.45	1705.469	5500.00	2449.490	0.227
Perioperative colloid use	729.03	382.268	750.00	534.522	0.919
Age	53.42	21.255	61.75	13.657	0.194
BMI	25.40	2.387	26.49	2.089	0.228
ASA status	1.77	0.560	2.00	0.535	0.314
Duration of anesthesia	454.03	117.597	568.75	93.417	0.012
Duration of surgery	443.39	115.732	556.88	91.844	0.017
Postoperative MAP	85.55	11.239	80.75	8.730	0.215
Postoperative lactate	1.65	0.972	1.76	0.607	0.677
Postoperative temperature	36.58	0.649	36.61	0.422	0.868
Amount of bleeding	811.29	839.018	581.25	322.864	0.233
Postoperative Ph	7.43	0.041	7.45	0.044	0.475
Postoperative Hb	11.79	1.919	10.77	1.004	0.132

**Table 4:** Comparison of parameters of patients with and without wound dehiscence.

Wound Dehiscense					
	Mean	SD	Mean	SD	*p*
Preoperative SPY score	65.38	26.483	71.04	17.046	0.456
Postoperative SPY score	73.11	27.964	52.91	23.408	0.074
Difference between preoperative and postoperative SPY scores	−7.73	8.542	18.13	23.976	0.001
Fluid balance for 48 h	1356.67	548.976	1245.56	727.961	0.680
Amount of perioperative transfusion	255.00	367.740	588.89	1137.095	0.410
Perioperative cristalloid use	4516.67	1966.633	4666.67	1802.776	0.833
Perioperative colloid use	736.67	386.392	722.22	506.897	0.939
Age	53.93	21.598	59.11	14.084	0.408
BMI	25.31	2.409	26.67	1.870	0.107
ASA status	1.77	0.568	2.00	0.500	0.253
Duration of anesthesia	469.50	126.541	504.44	104.177	0.415
Duration of surgery	457.83	125.125	496.11	99.114	0.354
Postoperative MAP	84.73	11.629	84.00	8.246	0.835
Postoperative lactate	1.61	0.967	1.88	0.653	0.345
Postoperative temperature	36.52	0.652	36.82	0.338	0.215
Amount of bleeding	673.33	426.237	1066.67	1407.347	0.431
Postoperative Ph	7.43	0.044	7.45	0.025	0.114
Postoperative Hb	11.64	1.800	11.40	1.928	0.745

**Table 5:** Comparison of the parameters of patients receiving postoperative mechanical ventilation support.

Mechanical Ventilation Support					
	Mean	SD	Mean	SD	*p*
Preoperative SPY score	67.28	32.293	66.39	20.361	0.916
Postoperative SPY score	74.93	35.819	65.21	23.384	0.314
Difference between preoperative and postoperative SPY scores	−7.65	6.247	1.18	20.202	0.135
Fluid balance for 48 h	1307.69	455,451	1342,69	650,074	0.863
Amount of perioperative transfusion	173.08	227.866	411.54	746.232	0.270
Perioperative cristalloid use	3600.00	1515.476	5026.92	1929.882	0.034
Perioperative colloid use	584.62	448.788	807.69	376.216	0.110
Age	49.46	23.365	57.96	18.025	0.217
BMI	24.86	2.369	26.01	2.282	0.153
ASA status	1.69	0.751	1.88	0.431	0.315
Duration of anesthesia	424.23	122.199	504.23	114.015	0.034
Duration of surgery	415.00	118.691	492.50	113.431	0.032
Postoperative MAP	84.69	15.124	84.50	8.305	0.959
Postoperative lactate	1.77	1.154	1.62	0.770	0.631
Postoperative temperature	36.69	0.496	36.53	0.655	0.450
Amount of bleeding	426.92	261.100	932.69	875.208	0.006
Postoperative Ph	7.42	0.048	7.45	0.035	0.093
Postoperative Hb	12.12	1.669	11.32	1.846	0.199

## Data Availability

No new data were created.

## References

[1] Tachon G., Harrois A., Tanaka S., Kato H., Huet O., Pottecher J., Vicaut E., Duranteau J. (2014). Microcirculatory alterations in traumatic hemorrhagic shock. Crit Care Med..

[2] Jones G.E., Garcia C.A., Murray J., Elwood E.T., Whitty A. (2009). Fluorescent intraoperative tissue angiography for the evaluation of the viability of pedicled TRAM flaps. Plast. Reconstr. Surg..

[3] Xie J., Mu R., Fang M., Cheng Y., Senchyna F., Moreno A., Banaei N., Rao J. (2021). A dual-caged resorufin probe for rapid screening of infections resistant to lactam antibiotics. Chem Sci..

[4] Zhang Y., Wang S., Sun Y., Xu H., Xu Z., Liang X., Yang J., Song W., Chen M., Fang M. (2024). Evaluation of a biomarker (NO) dynamics in inflammatory zebrafish and periodontitis saliva samples via a fast-response and sensitive fluorescent probe. Bioorg. Chem..

[5] Sjöberg T., Numan A., de Weerd L. (2021). Liberal versus Modified Intraoperative Fluid Management in Abdominal-flap Breast Reconstructions. A Clinical Study. Plast. Reconstr. Surg. Glob. Open.

[6] Gutierrez M.C., Moore P.G., Liu H. (2013). Goal-directed therapy in intraoperative fluid and hemodynamic management. J. Biomed. Res..

[7] Bennett V.A., Vidouris A., Cecconi M. (2018). Effects of Fluids on the Macro- and Microcirculations. Crit. Care.

[8] Cooper E.S., Silverstein D.C. (2021). Fluid Therapy and the Microcirculation in Health and Critical Illness. Front. Vet. Sci..

[9] Jansen S.M., de Bruin D.M., van Berge Henegouwen M.I., Strackee S.D., Veelo D.P., van Leeuwen T.G., Gisbertz S.S. (2017). Can we predict necrosis intra-operatively? Real-time optical quantitative perfusion imaging in surgery: Study protocol for a prospective, observational, in vivo pilot study. Pilot Feasibility Stud..

[10] Ibrahim A.M., Kim P.S., Rabie A.N., Lee B.T., Lin S.J. (2014). Vasopressors and reconstructive flap perfusion: A review of the literature comparing the effects of various pharmacologic agents. Ann. Plast. Surg..

[11] Kass J.L., Lakha S., Levin M.A., Joseph T., Lin H., Genden E.M., Teng M.S., Miles B.A., DeMaria S. (2018). Intraoperative hypotension and flap loss in free tissue transfer surgery of the head and neck. Head Neck.

[12] Grant D.W., Kim B.D., Halen J.P.V., Kim J.Y.S. (2014). Anesthesia Duration as an Independent Risk Factor for Postoperative Complications in Free Flap Surgery: A Review of 1305 Surgical Cases. J. Reconstr. Microsurg..

[13] Ettinger K.S., Arce K., Lohse C.M., Peck B.W., Reiland M.D., Bezak B.J., Moore E.J. (2017). Higher perioperative fluid administration is associated with increased rates of complications following head and neck microvascular reconstruction with fibular free flaps. Microsurgery.

[14] Wan M., Zhang J.X., Ding Y., Jin Y., Bedford J., Nagarajan M., Bucevska M., Courtemanche D.J., Arneja J.S. (2020). High-Risk Plastic Surgery: An Analysis of 108,303 Cases from the American College of Surgeons National Surgical Quality Improvement Program (ACS NSQIP). Plast. Surg..

[15] Ince C. (2015). Hemodynamic coherence and the rationale for monitoring the microcirculation. Crit. Care.

[16] Goncalves L.N., van den Hoven P., van Schaik J., Leeuwenburgh L., Hendricks C.H.F., Verduijn P.S., van der Bogt K.E.A., van Rijswijk C.S.P., Schepers A., Vahrmeijer A.L. (2021). Perfusion Parameters in Near-Infrared Fluorescence Imaging with Indocyanine Green: A Systematic Review of the Literature. Life.

[17] Dasta J.F., McLaughlin T.P., Mody S.H., Piech C.T. (2005). Daily cost of an intensive care unit day: The contribution of mechanical ventilation. Crit Care Med..

[18] Hackethal A., Hirschburger M., Eicker S.O., Mücke T., Lindner C., Buchweitz O. (2018). Role of Indocyanine Green in Fluorescence Imaging with Near-Infrared Light to Identify Sentinel Lymph Nodes, Lymphatic Vessels and Pathways Prior to Surgery—A Critical Evaluation of Options. Geburtshilfe Und Frauenheilkd..

[19] Massaro A., Gomez J., Weyh A.M., Bunnell A., Warrick M., Pirgousis P., Fernandes R. (2021). Serial Perioperative Assessment of Free Flap Perfusion with Laser Angiography. Craniomaxillofac. Trauma Reconstr..

[20] Chong M.A., Wang Y., Berbenetz N.M., McConachie I. (2018). Does goal-directed haemodynamic and fluid therapy improve peri-operative outcomes?: A systematic review and meta-analysis. Eur. J. Anaeesthesiol..

